# Non-Antioxidant Properties of α-Tocopherol Reduce the Anticancer Activity of Several Protein Kinase Inhibitors *In Vitro*

**DOI:** 10.1371/journal.pone.0036811

**Published:** 2012-05-08

**Authors:** Stéphane Pédeboscq, Christophe Rey, Muriel Petit, Catherine Harpey, Francesca De Giorgi, François Ichas, Lydia Lartigue

**Affiliations:** 1 INSERM U916 (VINCO), Institut Bergonié, Université Victor Segalen Bordeaux 2, Bordeaux, France; 2 Service Pharmacie, Hôpital Saint-André, CHU de Bordeaux, Bordeaux, France; 3 Fluofarma, Pessac, France; 4 Institut de Recherche Servier, Suresnes, France; Jawaharlal Nehru University, India

## Abstract

The antioxidant properties of α-tocopherol have been proposed to play a beneficial chemopreventive role against cancer. However, emerging data also indicate that it may exert contrasting effects on the efficacy of chemotherapeutic treatments when given as dietary supplement, being in that case harmful for patients. This dual role of α-tocopherol and, in particular, its effects on the efficacy of anticancer drugs remains poorly documented. For this purpose, we studied here, using high throughput flow cytometry, the direct impact of α-tocopherol on apoptosis and cell cycle arrest induced by different cytotoxic agents on various models of cancer cell lines *in vitro*. Our results indicate that physiologically relevant concentrations of α-tocopherol strongly compromise the cytotoxic and cytostatic action of various protein kinase inhibitors (KI), while other classes of chemotherapeutic agents or apoptosis inducers are unaffected by this vitamin. Interestingly, these anti-chemotherapeutic effects of α-tocopherol appear to be unrelated to its antioxidant properties since a variety of other antioxidants were completely neutral toward KI-induced cell cycle arrest and cell death. In conclusion, our data suggest that dietary α-tocopherol could limit KI effects on tumour cells, and, by extent, that this could result in a reduction of the clinical efficacy of anti-cancer treatments based on KI molecules.

## Introduction

Vitamin E was first described in 1922 as a dietary factor in animal nutrition [1]. It includes two groups (tocopherols and tocotrienols) of related fat soluble benzopyranol compounds, that are naturally found in vegetable oils [2]. It is also generally present as additive in industrially prepared foods and drinks and is declared in this case under the codes E306 to 309. In tocopherols, the C16 side chain is saturated while in tocotrienols, it contains three double bonds. Each group contains four constituents termed alpha (5,7,8-trimethyl), beta (5,8-dimethyl), gamma (7,8-dimethyl) and delta (8-methyl).

Since decades, vitamin E supplementation has been shown to possess beneficial effects for atherosclerosis, ischemic heart disease and cancer [3]–[5]. It is commonly assumed that these effects are linked to the antioxidant properties of these phenolic compounds [2], [6]. Vitamin E acts as a hydrophobic chain-breaking antioxidant that exerts a protective role against free radical damage to unsaturated lipids and hence membranes and tissues. Nevertheless, the sole antioxidant properties are presumably not sufficient to explain all the effects played by these molecules [2], and actually, in the past ten years, non-antioxidant functions of these agents have been described. Some authors have, for instance, reported pro-oxidant properties of α-tocopherol, showing that LDL peroxydation was faster in presence of this compound [7], [8]. Inhibition of PKC activity by α-tocopherol has also been shown to have a key role on vascular smooth muscle cell growth arrest [9], [10] and since, many papers have confirmed this effect on many other cell types such as monocytes, macrophages, neutrophils, fibroblasts and mesangial cells [11]–[14]. In activated human monocytes, α-tocopherol is also responsible for the decrease of proinflammatory cytokine IL-1β release via the inhibition of the 5-lipoxygenase pathway [15]. It is interesting to note that beta-tocopherol and trolox, contrary to α-tocopherol, do not possess these inhibitory properties [16], [17]. Gene expression could also be modulated by α-tocopherol, but not by beta-tocopherol. Some data have notably emphasized the ability of α-tocopherol to up-regulate the expression of tropomyosin gene [18] and down-regulate the expression of smooth muscle cells scavenger receptor gene (CD36), at the transcriptional level [19].

Beside these various properties, recent clinical trials on antioxidants supplementation have led to contradictory results. A meta-analysis by Bjelakovic et al. have showed, in particular, that beta-carotene, vitamin A or vitamin E supplementation does not have a beneficial effect on mortality but would rather increase the risk of death [20]. Even among vitamin E compounds, differences can be seen between tocotrienols and tocopherols. The former has indeed been shown to be a potent anticancer agent while the latter does not exhibit antitumoral activity even at extremely high concentrations [21]. The influence of antioxidants on the efficacy of anticancer treatments can thus be questioned since the induction of cell death by cytotoxic therapies usually holds an oxidative stress component that may be reduced by antioxidants. If it seems relatively intuitive for radiotherapy, the contribution of an oxidative stress in the mechanism of action of chemotherapeutic drugs used in clinic remains less clear. However, *in vitro*, the existence of a ROS-mediated amplification loop of cytotoxic effects has been evoked for different molecules such as DNA-damage inducing agents [22], TNF-α [23] or kinase inhibitors [24]. As α-tocopherol addition significantly reduced apoptosis induced by TNF-α, 7-ketocholesterol or nicotine in lymphoma or pulmonary epithelial cells, it was suggested that the protective role of the vitamin was a result of its anti-oxidative properties [21]. But unexpectedly, tocotrienols have opposite effects and potently induce apoptosis, suggesting that tocopherols and tocotrienols harbour distinctive intrinsic properties, and may act independently of their antioxidative capacities.

To better understand the mechanism of action of α-tocopherol, we conducted here a series of *in vitro* experiments specifically designed to monitor the real influence of α-tocopherol on the effects of various cytotoxic agents. We followed the effects of α-tocopherol addition on apoptosis and cell proliferation arrest induced by different agents. Our results show that α-tocopherol has an inhibitory effect on cytotoxic response, which is independent of its antioxidant potential.

## Results

### α-Tocopherol Inhibits Apoptosis Depending on Cell Death Inducers

**Figure 1:**
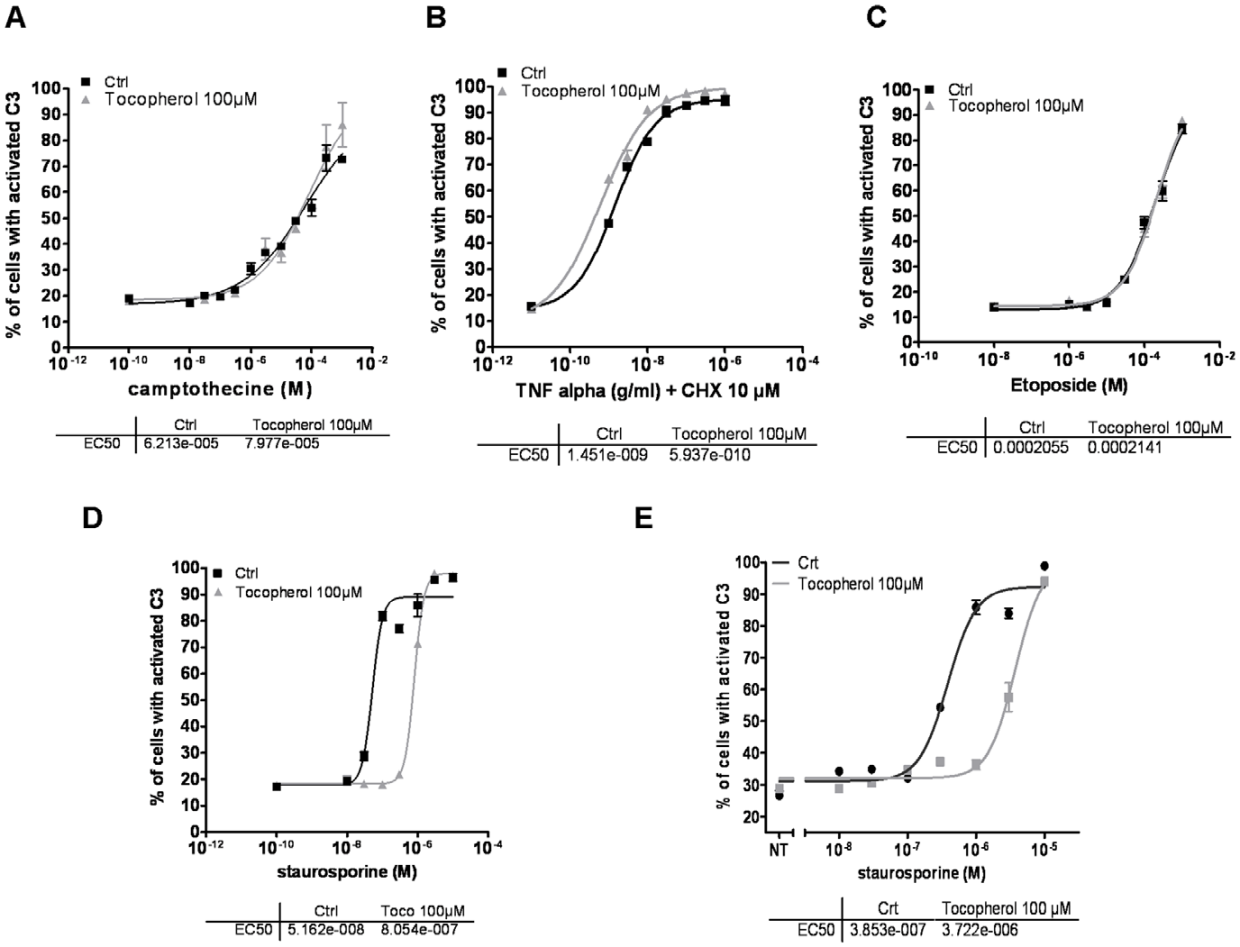
α-tocopherol inhibits staurosporine but not camptothecin, TNF-α or etoposide-induced apoptosis. In all cases, α-tocopherol was added simultaneously to drug treatment. **A–D** Dose response curves obtained for camptothecin (A), TNF-α supplemented with 10 µM of cycloheximide (CHX) (B), etoposide (C) and STS (D) in response to α-tocopherol treatment (100 µM) in a clone of HeLa cells stably expressing a caspase3/7-differential anchorage probe (C3/7-DAP). **E** Dose response of STS in a clone of DU145 (prostate carcinoma) expressing a C3/7-DAP was determined in presence or absence of α-tocopherol treatment (100 µM). In each case, EC_50_ were determined and are presented below the dose-response curves.

To elucidate which are the real repercussions of α-tocopherol addition on cell death, we determined the dose-response curves of different proapoptotic inducers in presence or not of the vitamin. Three different models of death-inducing pathways were tested in order to discriminate between a general or a specific effect of α-tocopherol on cell demise: camptothecin and etoposide were used as DNA damage inducers [25], [26], TNF-α the key mediator of inflammatory pathologies, was selected as a model of death-receptor mediated apoptosis and staurosporine (STS) was chosen as a pan kinase inhibitor, whose mechanism of action depends on the concentration of cellular exposure. Cell death was monitored using a GFP-based recombinant probe that measures the intracellular DEVDase activity, a specific marker of caspase-dependent apoptosis [27]. With this approach we evaluated the impact of antioxidant treatments specifically on the early steps of cell death. In HeLa cells, we found that the presence of α-tocopherol does not modify the dose-response curves of camptothecin, etoposide or TNF-αbut in contrast that it strongly inhibits cell death triggered by STS (Figure 1A–D). Indeed, α-tocopherol drastically reduces the percentage of caspase-3 positive cells post STS-treatment, increasing EC_50_ from 52 nM to 805 nM (Figure 1D). In addition, this protective effect of α-tocopherol against STS-induced cell death did not appear to be cell specific, as it was also observed on DU145 prostate cancer cells with a ten time increase in EC_50_ (Figure 1E).

### α-Tocopherol Protection from STS-induced Apoptosis does not Depend on its Antioxidant Properties

**Figure 2:**
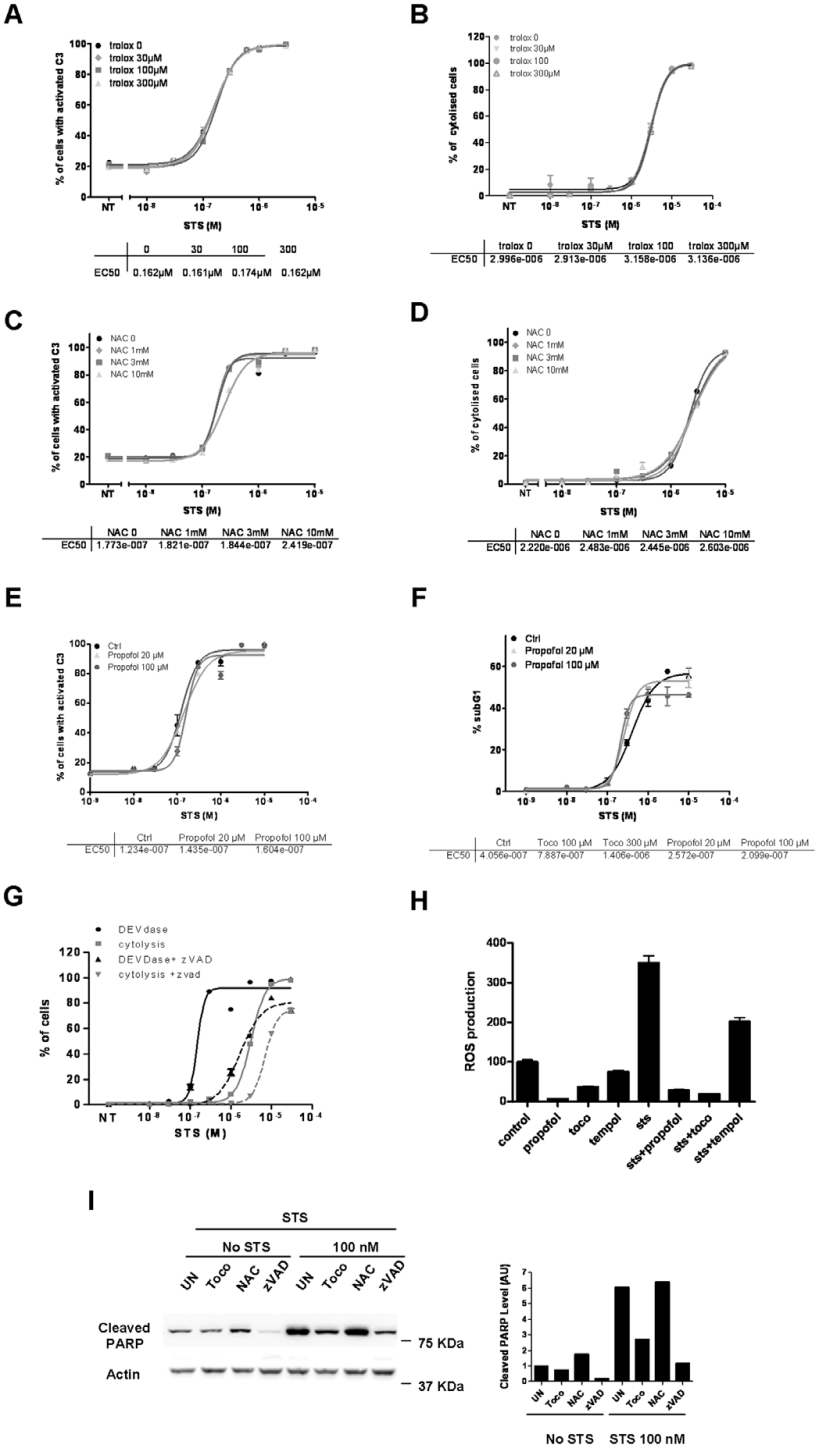
α-tocopherol protects from staurosporine-induced cell death independently of its antioxidant properties. When co-treatments were needed, antioxidants were added simultaneously to STS. **A, C, E** HeLa cells stably expressing a C3/7-DAP were treated with increasing amounts of STS in presence or not of different concentrations of trolox (A), NAC (C) or propofol (E). Dose response curves were established accordingly and EC_50_ values are presented below the curves. **B, D** HeLa cells were treated for 24 h with increasing amounts of STS in presence or not of trolox (B) and NAC (D) before measuring the percentage of cytolyzed cells using EMA staining. Dose response curves and EC_50_ values are shown below. **F** HeLa cells were treated with increasing amounts of STS in presence or not of propofol for 24 h. The percentage of subG1 cells was quantified after propidium iodide staining and dose response curves done accordingly. EC_50_ values are shown below. **G** DEVDase activity- and cytolysis- dose response curves in response to STS with or without the addition of zVAD, a pan caspase inhibitor. The number of cells was determined 24 h post-drugs treatment. **H** Quantification of ROS production in HeLa cells treated with vehicle or 30 nM of STS in presence or not of different antioxidants such as propofol (30 µM), α-tocopherol (100 µM), and tempol (100 µM) measured 24 hours after treatment using DCFDA as described in Materials and Methods. **I** Hela cells were treated with DMSO or 100 nM of STS in presence or not of 100 µM of α-tocopherol, 10 mM of NAC or 100 µM of zVAD for 24 hours. Apoptotic cell death was then evaluated by immunoblotting using an anti-cleaved PARP antibody. Actin was used as a loading control.

We first hypothesized that contrary to DNA damage or death receptor mediated cell death, STS toxicity would include a major oxidative stress component leading to caspase activation, and that this event would be blockable by the anti-oxidative properties of α-tocopherol. To verify this, we repeated the same experiments with three other antioxidants: trolox, NAC (N-acetyl cystein) and propofol (Figure 2). Contrary to α-tocopherol, trolox, a water-soluble vitamin E derivative, was not able to block STS-induced cell death. As presented in Figure 2A, the dose-response curves showing the percentage of cells with activated caspase-3 post-STS treatment perfectly fit in absence or in presence of three different concentrations of trolox (30, 100 and 300 µM). The same was observed with NAC (Figure 2C) and with propofol, a highly lipophilic anaesthetic drug that exhibits antioxidant potency similar to α-tocopherol (Figure 2E) [28]. Indeed, STS EC_50_ for caspase activation was neither modified after NAC nor after propofol addition. To confirm this intriguing result, we also evaluated the effect of trolox, NAC and propofol on later steps of apoptosis i.e on cell membrane permeability (cytolysis) (Figure 2B, 2D) and chromatin condensation (Figure 2F). Again, no protective effect of these antioxidants on cell death was observed (Figure 2A–F), while zVAD, a pan caspase-inhibitor, strongly reduced the percentage of STS-induced DEVDase activity or cytolysis (Figure 2G). This was also verified by western blot looking at PARP protein, a direct substrate of activated caspases. As shown in Figure 2I, α-tocopherol was as efficient as zVAD to block STS-induced PARP cleavage, contrary to NAC. Of note, zVAD alone also displayed some activity against the endogenous level of cleaved PARP, which indicates that a basal level of activated caspases resides in healthy cells. As α-tocopherol did not affect this background level of cleaved PARP, we concluded that this vitamin is certainly not a direct inhibitor of caspases (as also indirectly shown by the differential effect of α-tocopherol towards TNF-α, etoposide, camptothecin *versus* STS-induced cell death - in Figure 1). Finally, despite the ability of all the antioxidants tested here to reduce the production of ROS triggered by STS (Figure 2H), only α-tocopherol significantly blocked STS-mediated cell death, suggesting that this particular molecule can hold, under certain circumstances, potent anti-apoptotic properties that are independent of its antioxidant capacities.

### α-Tocopherol also Inhibits STS Effects on Proliferation and Cell Cycle Arrest

**Figure 3:**
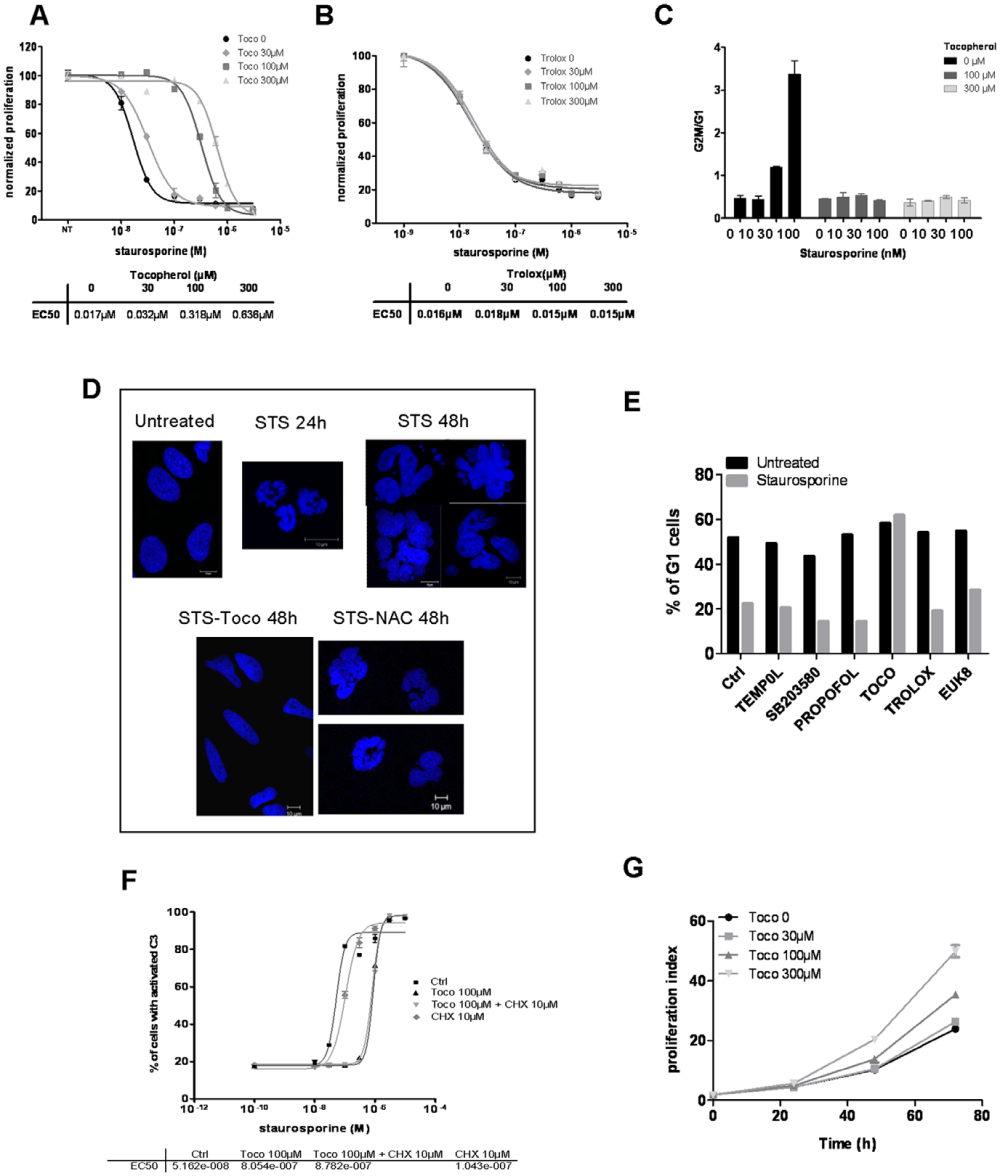
α-tocopherol prevents staurosporine-mediated cell cycle arrest in an antioxidant independent manner. A, B DiI-stained HeLa cells were treated with increasing amounts of STS supplemented with vehicle or 30, 100 and 300 µM of αtocopherol (A) or trolox (B) for 48 hours. Cell proliferation dose response curves were established from the median intensity fluorescence of DiI obtained for each point and normalized to the untreated control cells. EC_50_ values are shown in the table below the curves. **C** HeLa cells, co-treated with STS (1, 10, 30, 100 nM) and α-tocopherol (0, 100 or 300 µM) were stained with propidium iodide and cell cycle profile was determined by flow cytometry. The ratio of the percentage of cells in G2M on the percentage of cells in G0G1 was calculated and is shown here as a measure of the cell cycle profile variation. **D** HeLa cells were co-treated with 0.1 µM of STS and vehicle, α-tocopherol (100 µM) or NAC (3 mM) for 24 h or 48 h. Cells were fixed and colored with Hoechst. Images were acquired using the LSM510 confocal microscope. **E** HeLa cells were left untreated (black bars) or treated with 100 nM of STS for 24 h (grey bars). Antioxidants (100 µM of tempol, 10 µM of SB203580, 100 µM of propofol, 100 µM of α-tocopherol, 100 µM of trolox or 50 µM of euk8) were added simultaneously to STS. The percentage of cells in G0G1 phase was measured after propidium iodide staining and reported in the graph. **F** Dose response functions was obtained for STS in response to α-tocopherol treatment (100 µM) in presence or not of cycloheximide (CHX 10 µM) in a clone of HeLa cells stably expressing a C3/7-DAP. EC_50_ values were determined and presented in the table below the dose-response curves. **G** Growth curves of HeLa cells stained with DiI and treated with vehicle or 30, 100 and 300 µM of α-tocopherol for 24, 48 and 72 hours. Proliferation index was determined as described in the materials and methods section.

STS is a pan kinase inhibitor that can act on several protein kinases including those involved in cell cycle regulation. This drug is generally known as an apoptotic inducer but, at lower concentrations, it also leads to cell growth arrest. Accordingly, we found that, in HeLa cells, STS triggered an inhibition of cell proliferation with an EC_50_ of 17 nM (Figure 3A). In these conditions, α-tocopherol addition rendered cells far less sensitive to STS-mediated cell cycle arrest. As presented in Figure 3A, STS EC_50_ for cell proliferation increased in a dose-dependent manner in presence of the vitamin (EC_50_ was respectively multiply by 2, 20 and 40 in presence of 30, 100 and 300 µM of α-tocopherol compared to STS alone). On the contrary, the addition of another antioxidant, trolox, had no effect (Figure 3B).

We then stained HeLa cells for cell cycle profile and determined that STS provoked a blockade in G2/M phase that was overcome by α-tocopherol addition (Figure 3C). We finally showed that α-tocopherol prevented the accumulation of cells with condensed nuclei (at 24 h) or multilobes nuclei (at 48 h) that usually occurs in STS-treated samples due to the absence of cytokinesis following chromosomal replication (Figure 3D). As above, none of the other antioxidants tested (trolox, tempol, NAC, Euk8 and propofol) -nor the p38 inhibitor SB203580- were able to annihilate STS-induced cell cycle arrest (Figure 3B, D, E). Thus, as observed for apoptosis, α-tocopherol inhibited the antiproliferative properties of STS, while other antioxidants did not. To explore more in depth how α-tocopherol protected STS-treated cells from death, we sought to determine whether α-tocopherol effects were dependent on a transcriptional activity, as a direct action of α-tocopherol on gene expression has previously been described [18]. However, as shown in Figure 3F, the addition of cycloheximide, an inhibitor of protein synthesis, did not abrogate the ability of the vitamin to block STS-induced cell death, suggesting that the protective effect of α-tocopherol does not rely on a transcriptional mechanism, but is more direct. Of particular interest, it seemed that α-tocopherol was not only able to protect cells from STS-mediated apoptosis, but also to stimulate cell proliferation in a dose-dependent manner. HeLa cells proliferation index was, for instance, 2.5 times higher in a culture media supplemented with 300 µM of α-tocopherol than without (Figure 3G). So, it is possible that α-tocopherol engages proliferative pathways and stimulates them sufficiently enough to counteract the blockage of cell cycle triggered by STS treatment.

### α-Tocopherol Effects on other Kinase Inhibitors

**Figure 4:**
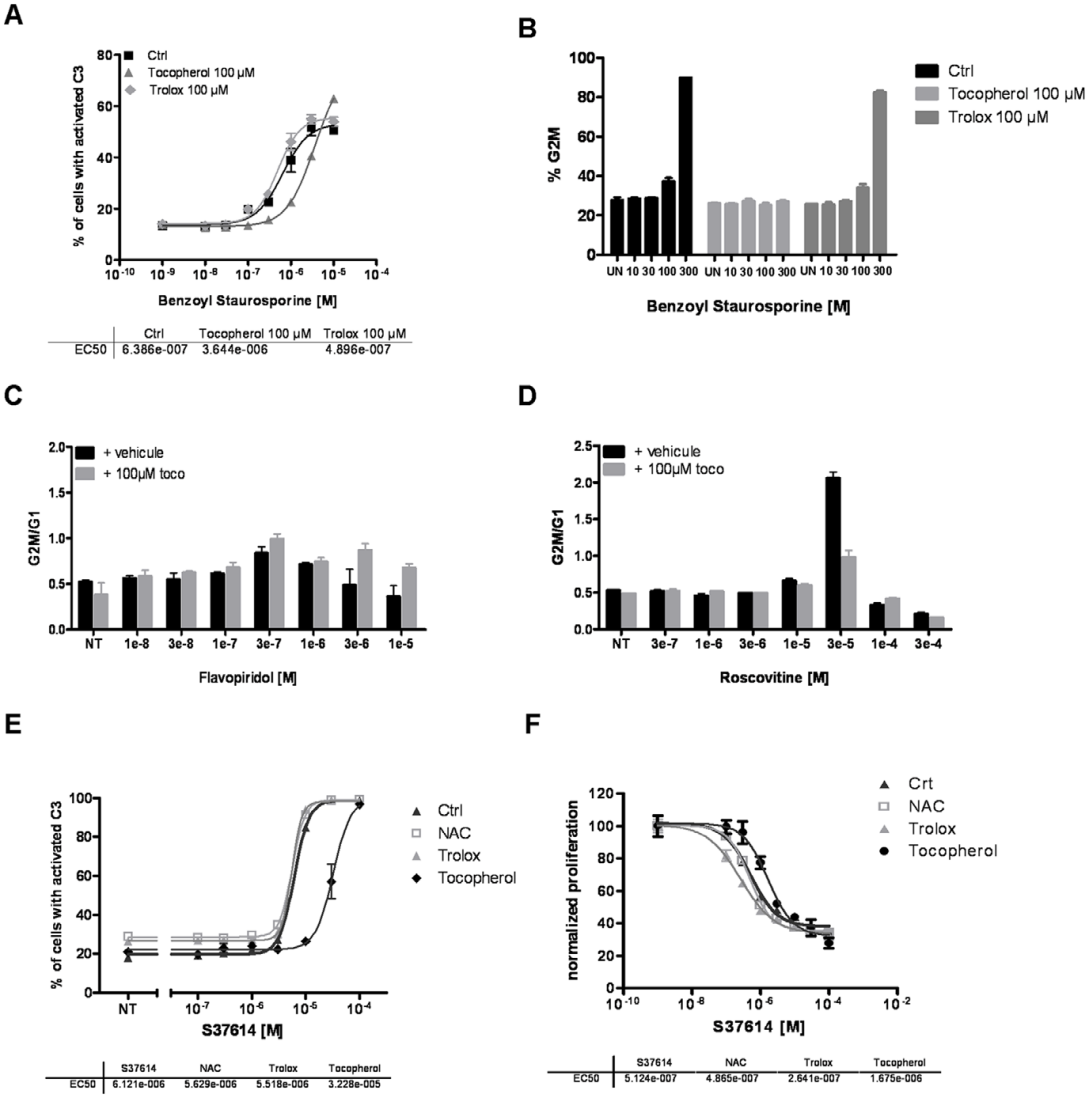
α-tocopherol blocks the anti-proliferative and pro-apoptotic properties of other kinase inhibitors independently of its antioxidant capacities. **A** Benzoyl-STS dose-response curves in presence or absence of 100 µM of α-tocopherol or 100 µM of trolox in a clone of HeLa cells stably expressing a C3/7-DAP. EC_50_ values are shown below. **B** HeLa cells were treated with various concentrations of benzoyl-STS in presence or not of 100 µM of α-tocopherol or 100 µM of trolox, and stained for cell cycle profile. The percentage of cells in G2M is reported here. **C, D** HeLa cells were treated with increasing amount of flavopiridol (**C**) or roscovitine (**D**) with or without 100 µM of α-tocopherol for 24 hours. Cell cycle profile was determined by flow cytometry using propidium iodide staining and the ratio of the percentage of cells in G2M on the percentage of cells in G0G1 was calculated and reported. **E** S37614 dose-response curves in presence or absence of 100 µM of α-tocopherol, 100 µM of trolox or 3 mM of NAC were determined using a clone of HeLa cells stably expressing a C3/7-DAP. **F** DiI-stained HeLa cells were treated with increasing amounts of S37614 supplemented with vehicle or 100 µM of tocopherol, 100 µM of trolox or 3 mM of NAC. Cell proliferation dose response curves were established from the median intensity fluorescence of DiI obtained for each point and normalized to the untreated control cells. EC_50_ values are shown in the table below the curves.

Our results show that α-tocopherol specifically blocks STS-induced cell death. In order to explore the specificity of this pharmacological interaction and determine if this effect can be generalized to other kinase inhibitors, we repeated the same experiments with several other drugs belonging to this class of molecules. We first analysed benzoyl-STS, a derivative of STS evaluated in phase I, phase II and one on-going phase III clinical trial [29], [30]. Similarly to STS, benzoyl-STS is a multi kinase inhibitor (PKC, VEGFR-2, PDGFR, KIT, MDR and FLT-3) [31] that triggers apoptosis in a dose-dependent manner, but with a higher EC_50_ (639 nM) (Figure 4A). As for STS, α-tocopherol exerted a significant protection against benzoyl-STS-induced apoptosis, increasing its EC_50_ by 6 times (EC_50_ = 3 600 nM). Trolox, at the opposite, had no effect (EC_50_ = 490 nM). At low concentrations, benzoyl-STS also induced a cell cycle arrest in G2/M that was totally prevented by the addition of α-tocopherol but not by trolox, exactly as observed with STS (Figure 4B). Two cell cycle kinases inhibitors were then studied: roscovitine and flavopiridol. Both drugs belong to the family of cyclin-dependent kinase (CDK) inhibitors and are under clinical development. While flavopiridol effects on cell cycle (that appeared to be moderate) did not seem to be sensitive toα-tocopherol, roscovitine dependent-G2/M blockade was highly reversed by α-tocopherol addition (Figure 4C and 4D). To finally ascertain that α-tocopherol was also efficient towards CDK inhibitors, we tested it on a new CDK inhibitor developed by Servier, named S37614. This drug, that preferentially targets CDK7, promotes caspase activation and apoptosis with an interesting EC_50_ (EC_50_ = 6.1 µM) (Figure 4E). Contrary to other antioxidants such as NAC (EC_50_ = 5.6 µM) and trolox (EC_50_ = 5.5 µM), α-tocopherol showed again a strong potential to protect cells that were committed to S37614-dependent cell death (EC_50_ = 32.3 µM) (Figure 4E). In addition, as described above, α-tocopherol also prevented S37614 effects on cell proliferation (Figure 4F). S37614 EC_50_ for cell proliferation was indeed multiply by 3 in presence of α-tocopherol, increasing from 513 nM to 1 675 nM. Other antioxidants did not exert such a protection: EC_50_ in presence of NAC, propofol or trolox being respectively equal to 487 nM, 520 nM and 264 nM. Thus, α-tocopherol exhibited an inhibitory effect on both S37614 pro-apoptotic and antiproliferative properties. Altogether, αtocopherol showed a strong inhibition against all kinase inhibitors tested except flavopiridol whose effect was weaker. These results, rather astonishing, underlie the non-negligible antagonist action of α-tocopherol on various kinase inhibitors, and raise the question of such consequences in a clinical context.

### α-Tocopherol Compromises the Effects of KI used in Clinical Practice

**Figure 5:**
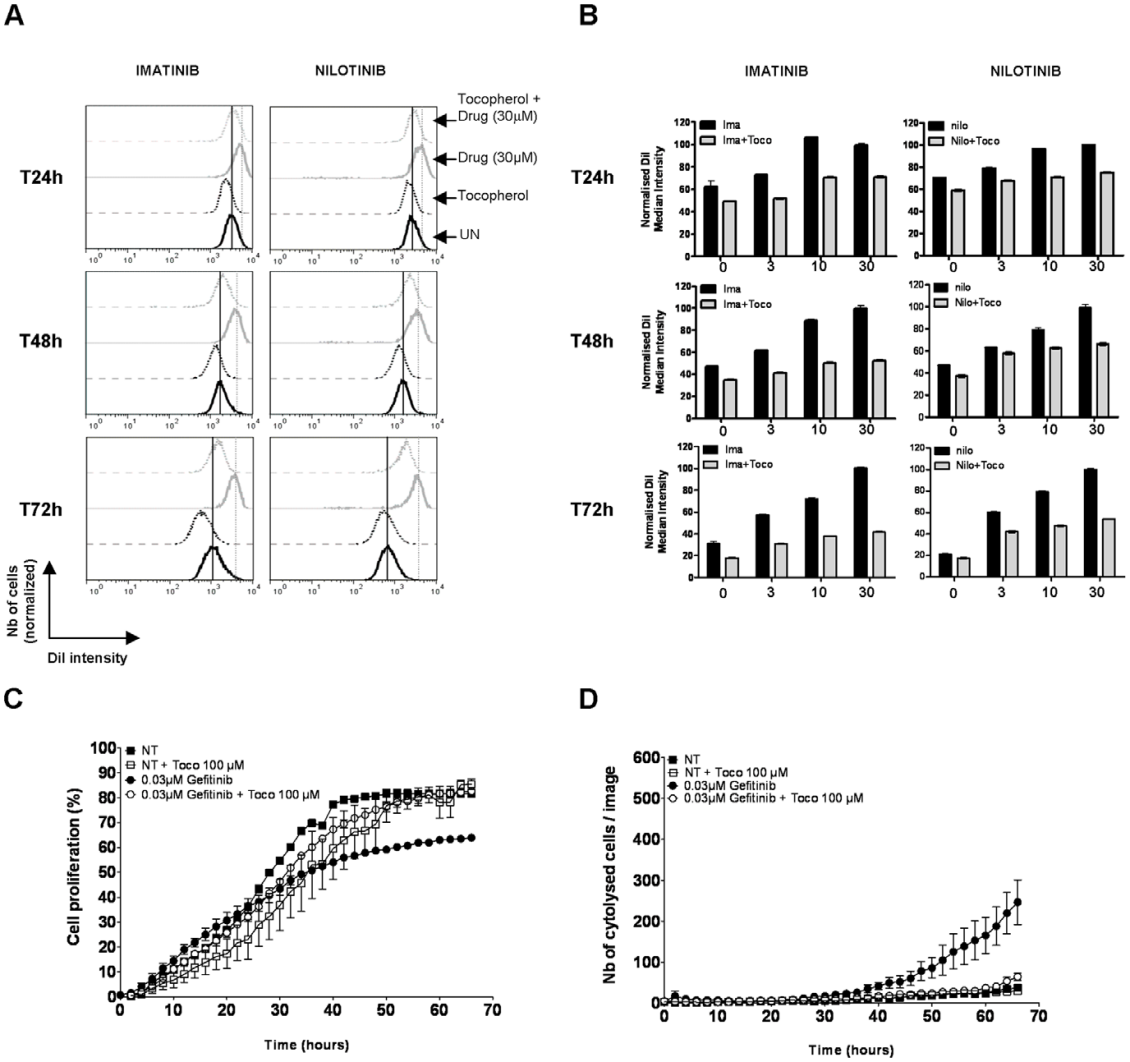
α-tocopherol prevents the action of kinase inhibitors used in clinical practice. A, B DiI-stained K562 cells were treated with increasing amounts of imatinib or nilotinib (0, 3, 10 or 30 µM) supplemented with vehicle or 1 µM of α-tocopherol. In each condition, cell proliferation was followed according to the intensity of DiI staining after 24, 48 and 72 hours. Cells exhibiting a brighter DiI fluorescence than control cells divide more slowly and are delayed in their cycle cell progression, while the ones having an equal or a weaker DiI intensity proliferate at the same rate or faster than control cells. An example of DiI fluorescence intensity post-imatinib or nilotinib treatment (30 µM) in presence or absence of 1 mM α-tocopherol is shown at each time point in **A**. The average DiI fluorescence intensity after all treatment is reported in panel **B** after normalization (100% correspond to the DiI most intense cells). **C** PC-9 cells were treated with DMSO or 0.03 µM of Gefitinib in presence or absence of 100 µM of α-tocopherol and immediately transferred to the IncuCyte system for kinetic cell growth measurement. The percentage of cell growth corresponds to the surface occupied by the cells in each well at each time point. **D** PC-9 cells were treated with DMSO or 0.03 µM of Gefitinib in presence or not of 100 µM of α-tocopherol, and incubated in presence of SYTOX Green in the IncuCyte system for cell death measurement.

To finally assess the effects of α-tocopherol addition on various cytotoxic drugs used in clinical practice, we chose to work with two different models of cancer cell lines that exhibit a relevant kinase-dependent transformed phenotype and for which protein kinase inhibitors are currently used as therapeutics. The first one corresponded to K562 cell line, a model of Chronic Myelogenous Leukemia (CML) characterized by the presence of the oncogenic BCR-ABL fusion protein that possesses a higher tyrosine kinase activity than the normal c-Abl protein and thus drives the aberrant proliferation and malignancy of these cells [32]. As an ideal candidate for targeted therapy, several drugs have been developed against BCR-ABL, among which the frontline therapy for CML, imatinib (or Gleevec) [33] and the second generation BCR-ABL inhibitors (also efficient on imatinib resistant form of BCR-ABL) nilotinib (Tasigna) [34], [35] and dasatinib (Sprycel) [36]. The second model corresponded to PC-9 cell line, a human lung adenocarcinoma driven by the oncogenic activation of the EGFR pathway caused by a deletion (Del E746–A750) in the exon 19 of the EGFR [37]. The presence of such a deletion has been found to be associated with a higher sensitivity to gefitinib (Iressa) or erlotinib (Tarceva), two selective EGFR kinase inhibitors [38], [39], [40]. We found that a co-incubation of α-tocopherol with imatinib or nilotinib prevented the anti-proliferative action of these drugs on K562 cells (Figure 5 A, B). Indeed, while the addition of 10 to 30 µM of imatinib or nilotinib induced a slow down of cell proliferation with a delay of one to two cell cycle(s) of division compared to control cells (DiI staining being, according the time point, 2 to 3 times less fluorescent in control cells than in imatinib or nilotinib cells), α-tocopherol supplementation significantly reverted this effect. In respect to that result, we also found that gefitinib’s action could be counteracted by the presence of α-tocopherol in PC-9 cells. Both the antiproliferative and cytotoxic activities of this KI were indeed reduced by the vitamin (Figure 5 C, D). Despite significant, the effects of α-tocopherol on these therapeutic compounds were slightly lower than for STS, probably due to their different way of action. Nonetheless, in certain conditions, α-tocopherol clearly delays or annihilates their anticancerous properties. Altogether, these data strongly suggest that α-tocopherol addition could, under certain circumstances of drug and vitamin concentrations, block the beneficial action of various kinase inhibitors used as novel targeted therapy in clinical practice, meaning that α-tocopherol could be critically involved in therapeutic failure.

## Discussion

α-tocopherol constitutes a vitamin E compound whose antioxidant properties are well known for decades. These interesting properties have led many investigators and clinicians to utilize this molecule in adjuvant supplementation diet to protect cells and tissues from free radical damage and oxidative stress. But recently, clinical trials on antioxidants supplementation have led to controversial results, showing that vitamin E, instead of being protective, would increase the risk of death [20], [41]–[43]. This states that the role of vitamin E is not totally understood and that some of its properties may counteract its protective effects. It also remains possible that vitamin E is more efficient as a preventive molecule rather than as a curative adjuvant. In that sense, we demonstrated here that α-tocopherol is able to inhibit the antiproliferative and proapoptotic effects of different chemotherapeutic agents and that this effect is not dependent on its antioxidant properties.

STS is a commonly used PKC inhibitor that is well known to trigger apoptosis in a large panel of cell lines [44]–[46]. In this work, we clearly showed that α-tocopherol inhibits, in a dose-dependant way, both the antiapoptotic and antiproliferative properties of STS. A similar observation has been described by a group in 2000 [47] on primary cultures of chick embryonic neurons. This study revealed a protective effect of tocopherol against STS-induced neuronal apoptosis, presumably due to the capacity of α-tocopherol to inhibit STS-induced glutathione stock depletion. Many authors have also demonstrated, in different cell types, that accumulation of ROS is a critical effector of STS-induced apoptosis [48], [49]. Under such circumstances, antioxidants addition would be thought to protect cells from an excessive production of ROS and consequently from apoptosis. So, to determine whether tocopherol action was dependent on its antioxidative properties, we evaluated the influence of other ROS scavengers on STS-induced apoptosis. We demonstrated that trolox, NAC, propofol and other antioxidants (Tempol and Euk8) were unable to reverse, contrary to α-tocopherol, the anticancer properties (both antiproliferative and apoptotic effects) of STS. So, the inhibitory capacities of α-tocopherol on this kinase inhibitor seem specific and cannot be extended to other antioxidants. Obviously, α-tocopherol has other intrinsic properties and may act in an antioxidant-independent manner. This is consistent with the fact that α-tocopherol, besides its well-known antioxidant function, possesses non antioxidant properties that have been explored by many other authors. Among them, the inhibition of PKC seems of particular importance, as it is also a target of STS. PKC possesses a regulatory (phospholipid and phorbol ester binding site) and a catalytic domain (peptide and ATP binding site) [46]. While STS is thought to be a direct ATP competitive inhibitor on the catalytic domain, α-tocopherol seems however to indirectly modulates PKC activity, by activating PP2A, an enzyme that specifically dephosphorylates and deactivates PKCα [6], [10], [50], [51]. By doing that, α-tocopherol would likely be able to interfere with STS action on PKC, even if added before STS and not necessary together with the vitamin. To verify this hypothesis, we evaluated the effect of α-tocopherol on STS-induced cell cycle arrest using two patterns of drugs treatment: in the first case (as all along the paper) STS and α-tocopherol were added simultaneously and, in the second case, a pre-treatment with αtocopherol alone was done before STS addition (not shown). Surprisingly, only the co-treatment with α-tocopherol was able to inhibit the cell cycle arrest induced by STS, while the pre-treatment with α-tocopherol was totally inefficient and unable to reverse the STS effect on cell cycle. These results would rather suggest a direct interaction between STS and α-tocopherol than a competitive effect of these compounds on PKC. Furthermore the ability of α-tocopherol to prevent the cytotoxicity of S37614 and roscovitine, that do not target PKC but CDK, reinforces this hypothesis.

**Figure 6:**
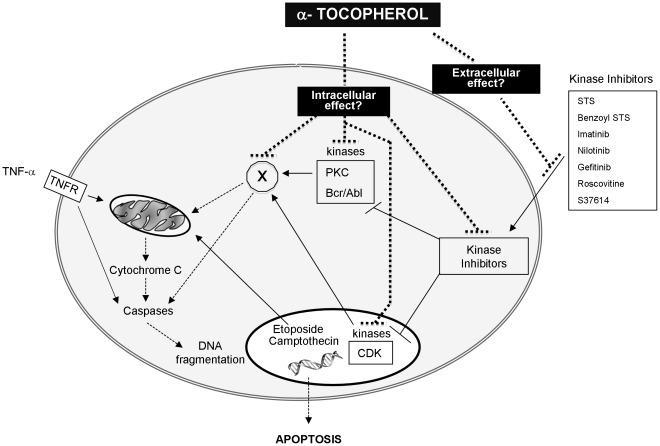
Schematic representation of the potential non-antioxidant properties of α-tocopherol on different pharmacological classes of anticancer drugs. Two hypotheses may be arisen in regards to the non-antioxidant protective role of α-tocopherol against the action of preclinical and clinical protein kinase inhibitors. The vitamin may either act outside the cells by preventing the different drugs to enter within the cell (extracellular effects) or inside by blocking the signalling pathway leading to death (intracellular effect). In the first case, we could imagine that α-tocopherol directly binds to the cytotoxic compounds or change the permeability of the cytoplasmic membrane to render it impermeable to this kind of inhibitors. In the second case, α-tocopherol would either directly act on (i) the kinase inhibitor, (ii) their targets or (iii) a common signalling partner engaged upon kinase inhibitors treatment and supposedly leading to death ( = “x” protein).

STS constitutes a standard apoptotic inducer, but cannot be used in clinical practice due to its non-selectivity and toxicity. For this reason, we also tested benzoyl-STS (known as PKC412 or midostaurin) an analog of STS, evaluated in clinical trials. Benzoyl-STS has similar effects on cell growth than STS but possesses a higher inhibitory specificity for PKC [29], [30]. We showed here that α-tocopherol exerts the same inhibitory properties against benzoyl-STS than STS. Another molecule, S37614 (Servier Laboratories) recently evaluated in preclinical trials, has been then tested to enlarge our panel of kinases inhibitors. As expected, S37614 inhibits cell growth of HeLa cell line. And, similarly to STS and benzoyl-STS, the anticancer effects of S37614 were counteracted by α-tocopherol. This vitamin, that showed a good potency to block CDK-inhibitors (S37614, roscovitin, flavopiridol), seems pretty efficient to hinder the effects of kinase inhibitors in general. This led us to extend our drug screening to kinase inhibitors used in clinical practice. This class of molecules has recently been widely spread with the introduction of targeted therapy in anticancer treatment protocols. We evaluated the repercussions of α-tocopherol addition on (i) imatinib and nilotinib treatment, two drugs used to inhibit the oncogenic fusion protein BCR-ABL in CML and (ii) on gefitinib effect, a well described EGFR inhibitor usually prescribed for non-small cell lung cancer. The results totally corroborate the data presented above, i.e. the presence of α-tocopherol in therapeutic regimen used in clinical practice blocks the antiproliferative effect of the drugs and compromises their beneficial action *in vitro*. To finally assess this specificity towards kinase inhibitors, we evaluated the influence of the vitamin on apoptosis induced by a few other anticancer drugs such as topoisomerase inhibitors and pro-apoptotic compounds such as TNF-α. We demonstrated that α-tocopherol had no effect and failed to protect cells from apoptosis induced by camptothecin and etoposide, respectively known as topoisomerase I and II inhibitors. And, identically, it has no repercussions on TNF-α induced cell death, despite that α-tocopherol has already been reported to protect U937 lymphoma cells form this kind of treatment, but for a short term period [52]. Altogether, our data indicate that α-tocopherol would preferentially target protein kinase inhibitors and impede their action. As it remains inactive towards etoposide, camptothecin or TNF-α, we hypothesized that its effect would rather occur in the upstream part of the death signalling pathway than in the downstream common part shared by many drugs triggering apoptosis. As displayed in Figure 6, we postulate that α-tocopherol may either act inside or outside the cell. In the first case, it may specifically block (i) the drug itself once incorporated into the cell, (ii) one of its targets or (iii) a common partner induced upon protein kinase inhibition, while in the second situation it would either change the permeability of cellular membrane to some drugs or directly bind to these latter. In both cases, this would prevent drugs from exerting their effects.

In conclusion, we demonstrated that α-tocopherol inhibits STS and more generally many kinase inhibitors anticancer effects, while other antioxidants failed to, suggesting that α-tocopherol engages different properties than its well-known antioxidant activity. These *in vitro* data also emphasize the putative negative effects of α-tocopherol supplementation on kinase inhibitors-based anticancer therapy.

## Materials and Methods

### Reagents

α-tocopherol, benzoyl-staurosporine, euk8, NAC (N-acetyl-cysteine), tempol and trolox were purchased from Calbiochem. Staurosporine, etoposide, camptothecin, zVAD (N-Benzyloxycarbonyl-Val-Ala-Asp(O-Me) fluoromethyl ketone), flavopiridol, cycloheximide, propofol, roscovitin and SB203580 were purchased from Sigma-Aldrich. TNF-α was from Invitrogen. imatinib, nilotinib and gefitinib were bought from Sequoia Research Product. S37614 kinase inhibitor was a generous gift from Servier’s Laboratory.

### Cells and Cell Culture

HeLa (Human cervical cancer cells, ATCC # CCL-2), DU145 (Human prostate carcinoma cells, ATCC # HTB-81) cell lines were grown in Dulbecco’s MEM medium (Gibco, Life technologies) supplemented with 10% fetal calf serum. K562 (Human chronic myelogenous leukemia cells, **#** CCL-243) and PC-9 (Human adenocarcinoma from lung tissue, ECACC # 90071810) cell lines were cultured in RPMI medium 1640 (Gibco, Life technologies) supplemented with 10% fetal calf serum. All cell lines were maintained at 37°C in a humidified atmosphere of 5% CO_2_/95% air. Plasmid transfection and generation of DAPs (Differential Anchorage probes) expressing stable clones were carried out as previously described [27].

### Differential Anchorage Probes Assays

The principle of this technique, allowing the detection of molecular events inside living cells such as caspase activation is described in details in a previous article [27]. Practically, DAP expressing cells were detached by trypsin, pooled and resuspended in an intracellular saline solution supplemented with 50 µM digitonin, 50 µM EGTA (ethylene glycol tetraacetic acid) and 4% fetal calf serum. Cells were then analyzed on a FACSCalibur flow cytometer (Becton Dickinson) equipped with an HTS (High Throughput Sampler) module and a 488-nm argon ion excitation laser. Fluorescence emission was measured in FL1 log mode.

### Determination of Cytolysis

To evaluate cytolysis, cells were incubated on ice with 100 µL of extracellular saline solution (NaCl 130 mM, KCl 3.6 mM, Hepes 10 mM, NaHCO_3_ 2 mM, NaH_2_PO_4_ 0.5 mM, MgCl_2_, 6H_2_O 0.5 mM, CaCl_2_, 2H_2_O 1.5 mM, Glucose 4.5 g/l) supplemented with 0.5 µg/mL ethidium monoazide bromide (EMA) for 10 min. Then cells were exposed to a white light for 15 min. Cells were then rinsed with 100 µL of extracellular saline solution for 5 min and with PBS for 5 min. Cells were detached with trypsin/EDTA and analysed by flow cytometry. EMA fluorescence was detected in FL3 channel on a FACSCalibur flow cytometer (Becton Dickinson) equipped with a 488-nm argon ion laser.

### Determination of Proliferation Index and Monitoring of Cell Proliferation (DiI Staining)

Before seeding, cells were incubated at 37°C in a solution of 5 µM DiI (1,1′-dioctadecyl-3,3,3′,3′-tetramethylindocarbocyanine perchlorate) and 0.3 M sucrose for 15 min under mild stirring. Cells were rinsed twice, counted and seeded in 96-well microtiter plates. The reference fluorescence value was measured at day 0 in FL3 channel using a FACSCalibur flow cytometer. The median intensity of DiI signal was then measured at day 1, 2 or 3. Values were either directly plotted or used to determine the proliferation index, corresponding to the ratio of DiI median intensity at day 0 on DiI median intensity at day 2 or 3.

### Flow Cytometric Analysis of Cell Cycle

The stock solution of propidium iodide was first diluted at 50 µg/mL in an intracellular saline solution (NaCl 10 mM; KCl 130 mM; Hepes 20 mM; MgSO_4_, 6H_2_O 1 mM; Succinate,6 H_2_O 5 mM) supplemented with 50 µM of digitonin and 50 µM of EGTA to permeabilize cellular membranes. After a pre-incubation of 5 min at room temperature, this mix solution was then added to the cells for 1 h at 4°C. PI signal was detected in FL3 channel by flow cytometry.

### ROS Level Measurement

ROS levels were quantified using a solution of dichloro-dihydro-fluoresceine-diacetate (H2DCFDA), as described in the manufacturer’s protocol (Invitrogen). Briefly, after trypsinisation, HeLa cells were incubated in presence of 20 µM of H2DCFDA. After 20 min at 37°C, ROS were quantified by flow cytometric analysis in FL1 channel using a FACSCalibur flow cytometer.

### Immunoblot Analysis

Hela cells were seeded in a 6 well-plate at 3×10^5^ cells/well and incubated with vehicle or 100 nM of STS in absence or presence of 100 µM of tocopherol, 10 mM of NAC or 50 µM of zVAD for 24 hours. Cells were then rinsed in PBS, pelleted and resuspended in 40 µl of lysis buffer (50 mM Tris-HCl, 150 mM NaCl, 1% NP-40, pH8) supplemented with a cocktail of protease inhibitors (Complete Mini-EDTA-free, Roche). After 1 hour at 4°C, lysed cells were spun down at 13 200 rpm for 15 min and all supernatants were collected. Protein concentration has been determined using the Bio-Rad Protein Assay kit (Bio-Rad laboratories GmbH, München, Deutschland). 50 µg of proteins were separated by electrophoresis on a 10% SDS polyacrylamide gel and transferred onto a PVDF (polyvinylidene fluoride) membrane (NEN Life Science Product, Boston, MA). Membrane was blocked 1 hour in PBST (phosphate buffered saline Tween 20) containing 5% low fat dry milk and probed with primary antibody diluted in TBST (Tris buffered saline Tween 20) with 5% BSA for anti-cleaved PARP antibody (1/1000; Cell Signaling, # 5625S) or PBST with 2.5% low fat dry milk for anti-actin antibody (1/1000; Sigma) overnight at 4°C. Membrane was then washed 4 times with TBST (for anti-PARP) or PBST (for anti-actin), incubated with horseradish peroxidase (HRP)-conjugated anti-rabbit secondary antibody (1/5000) for 1 hour at room temperature. After washing 4 times with TBST or PBST, protein bands were visualized using the Immobilon Western Detection System (Millipore) and quantified using ImageJ software.

### Imaging

Cells were seeded onto glass coverslips in 24-well plates the day prior drug treatment, and fixed 24 hours later with 3.7% formaldehyde for 10 min at room temperature (RT). After three washes in PBS, cells were incubated for 10 min with a solution of NH_4_Cl (50 mM) and immediately rinsed three times in PBS. Nuclear staining was done with a 1/5000 dilution of Hoechst 33258 (10 mg/ml) (Molecular Probes) for 10 min at RT. Coverslips were washed once in PBS and then mounted with Fluoromount G on glass slides. Image acquisition was performed using a Zeiss LSM 510 Meta microscope.

### Live-cell Kinetic Assays for Cell Proliferation and Cytolysis

5×10^3^ PC-9 cells were seeded into each well of a 96-well-plate and treated 24 hours later with gefitinib in presence or not of 100 µM of α-tocopherol. Cells were immediately transferred into the IncuCyte™ real-time imaging system (Essen Instruments) to follow kinetic cell proliferation and cytolysis. For cell growth measurement, high quality non-labelled phase-contrast images were acquired and automatically processed by a contrast-based confluence algorithm to determine the surface occupied by the cells in the well for each image at each time point. This value of cell confluence was then plotted to generate cell growth curves. For cytotoxicity measurement, cells were stained with SYTOX Green Nucleic Acid Stain (Invitrogen S7020) before being placed in the Incucyte system that also permits the acquisition of fluorescent images. The number of fluorescent cytolysed cells per image was determined for each image at each time point. In all cases, images were acquired every 2 hours for 70 hours.

## References

[1] Evans HM, Bishop KS (1922). On the Existence of a Hitherto Unrecognized Dietary Factor Essential for Reproduction.. Science.

[2] Ricciarelli R, Zingg JM, Azzi A (2001). Vitamin E: protective role of a Janus molecule.. Faseb J.

[3] Sigounas G, Anagnostou A, Steiner M (1997). dl-alpha-tocopherol induces apoptosis in erythroleukemia, prostate, and breast cancer cells.. Nutr Cancer.

[4] Pratico D, Tangirala RK, Rader DJ, Rokach J, FitzGerald GA (1998). Vitamin E suppresses isoprostane generation in vivo and reduces atherosclerosis in ApoE-deficient mice.. Nat Med.

[5] Keaney JF, Simon DI, Freedman JE (1999). Vitamin E and vascular homeostasis: implications for atherosclerosis.. Faseb J.

[6] Azzi A, Stocker A (2000). Vitamin E: non-antioxidant roles.. Prog Lipid Res.

[7] Bowry VW, Stanley KK, Stocker R (1992). High density lipoprotein is the major carrier of lipid hydroperoxides in human blood plasma from fasting donors.. Proc Natl Acad Sci U S A.

[8] Upston JM, Terentis AC, Stocker R (1999). Tocopherol-mediated peroxidation of lipoproteins: implications for vitamin E as a potential antiatherogenic supplement.. Faseb J.

[9] Boscoboinik D, Szewczyk A, Hensey C, Azzi A (1991). Inhibition of cell proliferation by alpha-tocopherol.. Role of protein kinase C. J Biol Chem.

[10] Tasinato A, Boscoboinik D, Bartoli GM, Maroni P, Azzi A (1995). d-alpha-tocopherol inhibition of vascular smooth muscle cell proliferation occurs at physiological concentrations, correlates with protein kinase C inhibition, and is independent of its antioxidant properties.. Proc Natl Acad Sci U S A.

[11] Freedman JE, Farhat JH, Loscalzo J, Keaney JF (1996). alpha-tocopherol inhibits aggregation of human platelets by a protein kinase C-dependent mechanism.. Circulation.

[12] Devaraj S, Li D, Jialal I (1996). The effects of alpha tocopherol supplementation on monocyte function. Decreased lipid oxidation, interleukin 1 beta secretion, and monocyte adhesion to endothelium.. J Clin Invest.

[13] Devaraj S, Adams-Huet B, Fuller CJ, Jialal I (1997). Dose-response comparison of RRR-alpha-tocopherol and all-racemic alpha-tocopherol on LDL oxidation.. Arterioscler Thromb Vasc Biol.

[14] Tada H, Ishii H, Isogai S (1997). Protective effect of D-alpha-tocopherol on the function of human mesangial cells exposed to high glucose concentrations.. Metabolism.

[15] Devaraj S, Jialal I (1999). Alpha-tocopherol decreases interleukin-1 beta release from activated human monocytes by inhibition of 5-lipoxygenase.. Arterioscler Thromb Vasc Biol.

[16] Martin-Nizard F, Boullier A, Fruchart JC, Duriez P (1998). Alpha-tocopherol but not beta-tocopherol inhibits thrombin-induced PKC activation and endothelin secretion in endothelial cells.. J Cardiovasc Risk.

[17] Cachia O, Benna JE, Pedruzzi E, Descomps B, Gougerot-Pocidalo MA (1998). alpha-tocopherol inhibits the respiratory burst in human monocytes. Attenuation of p47(phox) membrane translocation and phosphorylation.. J Biol Chem.

[18] Aratri E, Spycher SE, Breyer I, Azzi A (1999). Modulation of alpha-tropomyosin expression by alpha-tocopherol in rat vascular smooth muscle cells.. FEBS Lett.

[19] Ricciarelli R, Zingg JM, Azzi A (2000). Vitamin E reduces the uptake of oxidized LDL by inhibiting CD36 scavenger receptor expression in cultured aortic smooth muscle cells.. Circulation.

[20] Bjelakovic G, Nikolova D, Gluud LL, Simonetti RG, Gluud C (2007). Mortality in randomized trials of antioxidant supplements for primary and secondary prevention: systematic review and meta-analysis.. Jama.

[21] Sylvester PW (2007). Vitamin E and apoptosis.. Vitam Horm.

[22] Mizutani H, Tada-Oikawa S, Hiraku Y, Kojima M, Kawanishi S (2005). Mechanism of apoptosis induced by doxorubicin through the generation of hydrogen peroxide.. Life Sci.

[23] Temkin V, Karin M (2007). From death receptor to reactive oxygen species and c-Jun N-terminal protein kinase: the receptor-interacting protein 1 odyssey.. Immunol Rev.

[24] Sordet O, Khan QA, Plo I, Pourquier P, Urasaki Y (2004). Apoptotic topoisomerase I-DNA complexes induced by staurosporine-mediated oxygen radicals.. J Biol Chem.

[25] Pommier Y (2006). Topoisomerase I inhibitors: camptothecins and beyond.. Nat Rev Cancer.

[26] Baldwin EL, Osheroff N (2005). Etoposide, topoisomerase II and cancer.. Curr Med Chem Anticancer Agents.

[27] Schembri L, Zanese M, Depierre-Plinet G, Petit M, Elkaoukabi-Chaibi A (2009). Recombinant differential anchorage probes that tower over the spatial dimension of intracellular signals for high content screening and analysis.. Anal Chem.

[28] Gulcin I, Alici HA, Cesur M (2005). Determination of in vitro antioxidant and radical scavenging activities of propofol.. Chem Pharm Bull (Tokyo).

[29] Propper DJ, McDonald AC, Man A, Thavasu P, Balkwill F (2001). Phase I and pharmacokinetic study of PKC412, an inhibitor of protein kinase C. J Clin Oncol.

[30] Monnerat C, Henriksson R, Le Chevalier T, Novello S, Berthaud P (2004). Phase I study of PKC412 (N-benzoyl-staurosporine), a novel oral protein kinase C inhibitor, combined with gemcitabine and cisplatin in patients with non-small-cell lung cancer.. Ann Oncol.

[31] Vignot S, Soria JC, Spano JP, Mounier N (2008). [Protein kinases C: a new cytoplasmic target].. Bull Cancer.

[32] Ren R (2005). Mechanisms of BCR-ABL in the pathogenesis of chronic myelogenous leukaemia.. Nat Rev Cancer.

[33] Druker BJ (2004). Imatinib as a paradigm of targeted therapies.. Adv Cancer Res.

[34] Kantarjian H, Giles F, Wunderle L, Bhalla K, O’Brien S (2006). Nilotinib in imatinib-resistant CML and Philadelphia chromosome-positive ALL.. N Engl J Med.

[35] Weisberg E, Manley PW, Breitenstein W, Bruggen J, Cowan-Jacob SW (2005). Characterization of AMN107, a selective inhibitor of native and mutant Bcr-Abl.. Cancer Cell.

[36] Shah NP, Tran C, Lee FY, Chen P, Norris D (2004). Overriding imatinib resistance with a novel ABL kinase inhibitor.. Science.

[37] Bean J, Brennan C, Shih JY, Riely G, Viale A (2007). MET amplification occurs with or without T790M mutations in EGFR mutant lung tumors with acquired resistance to gefitinib or erlotinib.. Proc Natl Acad Sci U S A.

[38] Lynch TJ, Bell DW, Sordella R, Gurubhagavatula S, Okimoto RA (2004). Activating mutations in the epidermal growth factor receptor underlying responsiveness of non-small-cell lung cancer to gefitinib.. N Engl J Med.

[39] Paez JG, Janne PA, Lee JC, Tracy S, Greulich H (2004). EGFR mutations in lung cancer: correlation with clinical response to gefitinib therapy.. Science.

[40] Pao W, Miller V, Zakowski M, Doherty J, Politi K (2004). EGF receptor gene mutations are common in lung cancers from “never smokers” and are associated with sensitivity of tumors to gefitinib and erlotinib.. Proc Natl Acad Sci U S A.

[41] Stanner SA, Hughes J, Kelly CN, Buttriss J (2004). A review of the epidemiological evidence for the ‘antioxidant hypothesis’.. Public Health Nutr.

[42] Berger MM (2005). Can oxidative damage be treated nutritionally?. Clin Nutr.

[43] Bjelakovic G, Nikolova D, Simonetti RG, Gluud C (2004). Antioxidant supplements for prevention of gastrointestinal cancers: a systematic review and meta-analysis.. Lancet.

[44] Ishizaki Y, Voyvodic JT, Burne JF, Raff MC (1993). Control of lens epithelial cell survival.. J Cell Biol.

[45] Jarvis WD, Turner AJ, Povirk LF, Traylor RS, Grant S (1994). Induction of apoptotic DNA fragmentation and cell death in HL-60 human promyelocytic leukemia cells by pharmacological inhibitors of protein kinase C. Cancer Res.

[46] Gescher A (1998). Analogs of staurosporine: potential anticancer drugs?. Gen Pharmacol.

[47] Ahlemeyer B, Krieglstein J (2000). Inhibition of glutathione depletion by retinoic acid and tocopherol protects cultured neurons from staurosporine-induced oxidative stress and apoptosis.. Neurochem Int.

[48] Kruman I, Guo Q, Mattson MP (1998). Calcium and reactive oxygen species mediate staurosporine-induced mitochondrial dysfunction and apoptosis in PC12 cells.. J Neurosci Res.

[49] Coyoy A, Valencia A, Guemez-Gamboa A, Moran J (2008). Role of NADPH oxidase in the apoptotic death of cultured cerebellar granule neurons.. Free Radic Biol Med.

[50] Azzi A, Boscoboinik D, Chatelain E, Ozer NK, Stauble B (1993). d-alpha-tocopherol control of cell proliferation.. Mol Aspects Med.

[51] Chatelain E, Boscoboinik DO, Bartoli GM, Kagan VE, Gey FK (1993). Inhibition of smooth muscle cell proliferation and protein kinase C activity by tocopherols and tocotrienols.. Biochim Biophys Acta.

[52] Yano M, Kishida E, Iwasaki M, Kojo S, Masuzawa Y (2000). Docosahexaenoic acid and vitamin E can reduce human monocytic U937 cell apoptosis induced by tumor necrosis factor.. J Nutr.

